# Possible Roles of Permafrost Melting, Atmospheric Transport, and Solar Irradiance in the Development of Major Coronavirus and Influenza Pandemics

**DOI:** 10.3390/ijerph18063055

**Published:** 2021-03-16

**Authors:** Anne M. Hofmeister, James M. Seckler, Genevieve M. Criss

**Affiliations:** 1 Department of Earth and Planetary Science, Washington University, St. Louis, MO 63130, USA; 2 Department of Biomedical Engineering, Case Western Reserve University, Cleveland, OH 44106, USA; jms92@case.edu; 3 Webster Central School District, Webster, NY 14526, USA; genevieve_criss@websterCSD.org

**Keywords:** COVID-19, historic influenzas, permafrost melting, pandemic emergence, climate and disease, ultraviolet immunosuppression, environment and health, airborne transport

## Abstract

Major pandemics involving respiratory viruses develop semi-regularly and require a large flux of novel viruses, yet their origination is equivocal. This paper explores how natural processes could give rise to this puzzling combination of characteristics. Our model is based on available data regarding the emergence of historic influenzas, early COVID-19 cases and spreading, the microbiome of permafrost, long-distance airborne transport of viruses reaching stratospheric levels, ultraviolet immunosuppression, sunlight variations, weather patterns, Arctic thawing, and global warming. Atmospheric conveyance is supported by hemispheric distribution disparities, ties of COVID-19 cases to air pollution particulate concentrations, and contemporaneous animal infections. The following sequence is proposed: (1) virus emergence after hot Arctic summers, predominantly near solar irradiance maxima or involving wildfires, indicates release of large amounts of ancient viruses during extensive permafrost melting, which are then incorporated in autumn polar air circulation, where cold storage and little sunlight permit survival. (2) Pandemics onset in winter to spring at rather few locations: from climate data on Wuhan, emergence occurs where the North Polar Jet stream hovers while intersecting warmer, moist air, producing rain which deposits particulates with the viral harvest on a vulnerable human population. (3) Spring and summer increases in COVID-19 cases link to high solar irradiance, implicating ultraviolet immune suppression as one means of amplification. (4) Viruses multiplied by infected humans at close range being incorporated in atmospheric circulation explains rapid global spread, periodic case surges (waves), and multi-year durations. Pollution and wind geography affect uptake and re-distribution. Our model can be tested, e.g., against permafrost stored in laboratories as well as Artic air samples, and suggests mitigating actions.

## 1. Introduction and Background

Influenza and coronaviruses are the root cause of many pandemics and epidemics. Communication at short range (<2 m) between humans predominantly via respiration adequately explains epidemics, which are localized with varying intensity and have occurred frequently over history. However, conditions permitting development of the much rarer, major respiratory virus pandemics (MRVPs) are not well understood [1]. Models of origination are incomplete because detailed investigations are limited to either recent pandemics, which were comparatively minor, or to large, historical events such as the 1918 influenza for which essential information is unavailable and/or inaccurate, being gathered in retrospect. In view of the information stream on the ongoing COVID-19 pandemic and recent studies on diverse aspects of viruses, two basic assumptions regarding pandemic emergence merit reconsidering, as follows:

High impact (>10% infected) on a global scale requires a long-range mechanism for spreading. However, the current assumption, that international jet travel facilitates human conveyance, does not address several observations:

(1) Invention of airplanes has not hastened onsets: early cases of the 1889 influenza pandemic appeared in St Petersburg 10 days before eruption in London and other European cities, and only 5 weeks prior to onset in New York [2]. This timing outpaces initial development of COVID-19, where the first appearance in China preceded surges in Italy and Iran by ~5 weeks and the US by ~9 weeks. Instead, changes in spreading rates within any given country of MRVPs over the past ~230 years, as presented by Sanders-Hasting and Krewski [1], strongly correlate with advances in ground transportation. Thus, more than one mechanism of spreading exists.
(2) Although jet travel dispersed the first SARS coronavirus in 2003, it only provided 1–3 cases in many countries, whereas >7500 out of ~8100 total cases occurred in China or nearby [3]. Since below 0.0002% of the global population was infected, SARS-2003 is best described as an epidemic with outliers, and indicates that jet travel is insufficient to produce large numbers of infections worldwide.
(3) Location of COVID-19 case surges in June 2020 across the southernmost US, but not in the northern and eastern cities which drew large crowds of protestors in May 2020, strongly suggests that factors besides human proximity cause spreading.
(4) Viruses with very high genetic identity are found in very distant and different environments, which can be explained by atmospheric transport of organic aerosols [4]. Rapid distribution of high volumes of viruses over large distances characterizes pandemics. This can be realized through airborne transport, and is considered as the mechanism for the annual minor influenza pandemic, after Hammond et al. [5]. Viruses in general are transported and transmitted via aerosols [6]. Section 1.2 summarizes data on atmospheric transport of viruses.

Discussions of MRVP emergence implicitly assume spreading of infections from one specific locality, initially involving rather few individuals. In the case of COVID-19, it has been suggested that it originated in either a wet marketplace in Wuhan, China or via an accidental release from a Wuhan laboratory. Both hypotheses are disputed [7,8], which has motivated proposals of alternatives and calls for greater autonomy in investigations [9,10]. Thus, the source of the novel SARS-CoV-2 virus which causes the COVID-19 disease remains a key issue. Previous microbiology studies of permafrost (summarized in Section 1.1) motivated us to consider this environment as an alternative to a laboratory or wet marketplace origin. 

As in other MRVPs, “the outbreak would appear to suddenly come out of nowhere” [11]. On this basis, not only are the physical locality and nature of the source for the “novel” coronavirus relevant to understanding emergence, but equally important is how the virus is transported from whence it originated to large segments of the human population. Respiratory viruses are primarily spread through inhalation. This route underlies social distancing, since the amount of viruses exhaled from an infected person decreases with distance. Dilution occurring via lateral spread is indicated by closed circulation of air, as in an air-conditioned automobile, counteracting physical separation [12]. A model for the origin of the COVID-19 pandemic should thus focus on uptake via respiration which involves airborne transport of the virus over various distances. 

To construct a model for development of MRVPs, some additional factors warrant consideration. COVID-19 case surges are seasonal, which also characterizes influenzas, but is incompletely understood [13]. The sporadic, but repeated occurrence of MRVPs through history is extremely important to causation. Because semi-regular appearance of MRVPs has not been explained by etiologic and epidemiology studies [14], we propose that natural phenomena, specifically those connecting humans with the environment, play key roles in pandemic development. Our investigation is based on the above discussion, and on information provided by additional diverse studies of viruses, summarized in Section 1.1, Section 1.2, Section 1.3 and Section 1.4. These previous efforts point to several natural processes worth considering.

### 1.1. Permafrost as a Possible Source of Large Amounts of Novel Viruses

The appearance of a viral strain for which many humans lack immunity underlies historic and modern MRVPs. Melting of ~30,000-year-old permafrost can release trapped viruses and thus constitutes a potential health hazard [15]. Although techniques used by these authors cannot resolve the small sizes of respired viruses, permafrost (PF) microbes are diverse and abundant, and their release following warming episodes has caused bacterial infections in circumpolar regions [16,17]. Soil viruses released during thaws are important contributors to carbon cycling [18]. Importantly, summer warming increases the number and diversity of viruses in PF thaw lakes [19]. 

An Arctic or sub-Arctic source is consistent with major and minor RVPs being predominantly a Northern Hemisphere (NH) phenomenon [1,14]. This potential reservoir is immense, ~10^17^ kg, rich in organics [15,16,17], and dwarfs the entire mammalian biomass. Mutation from an animal source such as bats or a laboratory accident are currently popular hypotheses, but are controversial [7,8,9,10]. Importantly, neither proposal for the origin addresses the semi-regular appearance of respiratory pandemics at approximately ~50 year intervals. In addition, viruses from bats in Thailand, Cambodia, and Japan are now known to be similar to SARS-CoV-2 [20,21]. A dispersed precursor seems incompatible with explosive emergence over a short time period at a restricted locality. Moreover, as pointed out by Wacharapluesadee et al. [20], an immediate animal ancestor or progenitor virus that is ~99% identical to SARS-CoV-2 has not been found.

Viruses stored in permafrost for millennia are “new” to organisms now alive. Recent appearance of three heretofore unknown coronaviruses synchronizes with the unprecedented high rates of current global warming and permafrost melting. Hence, comparing climate changes with MRVP timing is warranted. 

### 1.2. Long-Distance Atmospheric Transport of Microbes, Including Coronaviruses

Continuous exchange through the atmosphere of large amounts of diverse microorganisms among ecosystems over thousands of km, including between continents [22], makes the airborne microbiome important to human health. In a 7 year study, Caliza et al. [23] identified freshwater, cropland, and urban biomes as the most important sources for airborne bacteria in summer, whereas marine and forest biomes prevailed in winter, along with higher concentrations of potential pathogens. These differences were linked to seasonal variations in general and regional atmospheric circulation, in sources of the aerosols, and in environmental factors. These findings are supported by subsequent research (e.g., [24]).

Viruses are also airborne, including those associated with COVID-19, since its RNA was detected in Northern Italian air ([25]; see Section 1.3). Generally speaking, viruses in aerosols are deposited at rates substantially larger than rates for bacteria and are associated with smaller organic aerosol sizes (<0.7 μm) than observed for bacteria [4]. Their observations indicate that viruses have longer residence times in the atmosphere and thus will travel further, which explains why distant and different environments contain viruses with very high genetic identity (e.g., [26]). Reche et al. [4] further demonstrated that viruses exist above the atmospheric boundary level (~3 km altitude in the Spanish Sierra Nevada Mountains) which is in the free atmosphere part of the troposphere and thereby enables long-distance transport. Much greater heights are possible, as indicated by collection of bacteria from the stratosphere which resemble those in the troposphere [27], although viability decreases above the tropopause at 12 km. Organic aerosols protect viruses against ultraviolet (UV) radiation [28], which assistslive viruses surviving global transport in the strong winds of the troposphere. 

If the physical source of novel viruses is indeed the Arctic, then subsequent transport southwards in the NH is required to start a pandemic. Long distance travel from permafrost is confirmed by the study of Girard et al. [19] who found highly similar viral genomes in three freshwater Wisconsin bog lakes which lie 1600 km SW of their Canadian PF study site. Wuhan lies 2000 km south of widespread permafrost in Mongolia. Wuhan is an urban wetland with 138 lakes and marshes, whose ecological function has been greatly disturbed in recent decades [29]. Marshes differ from bogs by being much richer in nutrients due to more rapid decay. The surrounding Hubei province contains Dajiuhu National Wetland which is the largest and highest wetland in central China and consists of subalpine sphagnum bog [30]. The similarity of the North American and Asian situations supports considering PF as the source of COVID-19, in view of the above documentations of long-distance and high-level atmospheric transportation.

Furthermore, historic influenza MRVPs have emerged seasonally in rather few geographic locations, often near China or Russia, even though tracing is difficult [1]. This observation is consistent with seasonal release of viruses from permafrost (Section 1.1) and further suggests influence by global weather patterns, which are geographically controlled (climate zones). 

Based on the above findings, our paper compares copious data on atmospheric phenomena to the development, geographic distribution, and subsequent spread of COVID-19.

### 1.3. Roles of Particulates and Pollution in Virus Transport and COVID-19 Cases

Airborne microorganisms can be removed from the atmosphere by rain or by direct sedimentation. Association of COVID-19 cases with air pollution particulates thus bears on its emergence.

Regions with heavy industrial development (Wuhan and Northern Italy) experienced aggressive spread of the virus (e.g., [6]). Disagreement on the precise nature of the correlation in early studies is attributable to pollution particulates being a known health hazard by themselves [31]. Recently, Hutter et al. [32] showed that air pollution in 23 different districts within a single city (Vienna, Austria) correlated with COVID-19 cases and mortality. By considering one locality, this study minimized the number of variables and thus definitively linked this respiratory disease with increased concentrations of airborne particulates. 

Direct measurements of particulates in the air in Northern Italy by Setti et al. [25] revealed the presence of RNA associated with COVID-19. The correlation between outdoor atmospheric pollutants and COVID-19 infections was subsequently demonstrated by several studies while a few studies have shown a connection of the disease with electrostatically charged fine particles indoors [33]. Soot, which is amorphous carbon produced during combustion, is a type of electrostatically charged particle.

In the Arctic summers, wildfires are common, which produce soot from organics in the soil. Surface fires can be extinguished, but buried peat and methane deposits permit subsurface smoldering throughout the cold and wet Arctic winters [34]. Spontaneous surface eruptions follow in the spring, as the snow blanket sublimates, resulting in “zombie” fires, which also supply organic carbon particulates (and therefore microbes) to the atmosphere. Importantly, summer 2019 was a record-setting Arctic fire season [35], which was surpassed in summer 2020 through re-ignition [36]. These record-setting wildfires align with record-setting high summer temperatures [37] which promote PF melting, discussed further in Section 3.2. Importantly, melting of permafrost is now escalating, which is of great concern for other reasons (e.g., [38]). 

Warmth from subsurface fires promotes year-round melting of adjacent PF, whereas the particulates and updrafts from the above-ground fires provide a mechanism for ascent of viruses released in the summers, which can then be dispersed in atmospheric currents (Section 1.2). Descent of particulates is provided by rain, as is well known from observations of wildfires and of smoggy locations. Dry descent of viruses also occurs [4].

The above observations support considering PF as the source, and atmospheric transport as the distribution mechanism.

### 1.4. Effect of Solar Ultraviolet Light on Temperature, Viruses, and Immune Response

Subsequent to the June 2020 growth in the US Sunbelt, cases increased in other sunny locations, such as Spain, despite restrictions on travel. Thus, we compare variations in sunlight with respect to time and place to data on MRVPs, based on human immune response being compromised by high ultraviolet (UV) influx [39,40,41,42,43] (see Section 5.3). However, we terminate our analysis of case number in July 2020 because the subsequent increase in regional and global travel likely overprinted data relevant to the how pandemics begin, which is the topic of our paper. 

We also consider periodicity of sunlight variations and of temperature, particularly in the Arctic, since seasonality plays a role in influenzas and coronaviruses, as well as in PF melting, and because the semi-periodicity of pandemics calls for an explanation. 

### 1.5. Hypothesis and Organization of the Paper

Our hypothesis—that certain natural environmental processes, coupled with human response to UV radiation, underlay development of MRVPs—is simple. However, because both MRVP development and natural processes are complex, we begin with a synopsis of our model: Table 1 summarizes the several stages and various environmental processes involved. Stages are required, as is clear from the “waves” in the 1918 influenza, the seasonal nature of yearly influenzas which are minor pandemics, and temporal variations of COVID-19 cases, which continue today. Our model of Table 1 was deduced by considering results from previous studies of respiratory viruses (Section 1.2, Section 1.3 and Section 1.4) and by comparing various datasets (described in Section 2). Evidence for the stages described in Table 1 is presented in Section 3, Section 4 and Section 5. Section 6 summarizes the model. Section 7 discusses how this proposal can be tested beyond the evidence presented here, and covers implications of our findings. Section 8 briefly summarizes.

## 2. Available Data and Methods

To explore emergence, the focus is COVID-19 cases up to July 2020, which are archived; see [44,45,46]. Data presented in the figures are publicly available and were downloaded from various sources, as noted. Using compilations is necessary since our comparison involves ~140 countries reporting data on COVID-19. Uncertainties in the numbers of cases, particularly for underdeveloped countries and early on, in the numbers of people infected but not tested worldwide, and in the early tests themselves, are large. We also used compilations on the web to ascertain other data for countries, e.g., the gross domestic product, population, or land area. Trends in the data are presented graphically. Least squares fits were used. For geographical information, either Google maps or Google Earth was used. Additional data sources and other resources are noted in the text or figures.

## 3. Permafrost Melting as the Source of Pandemic-Generating Viruses

### 3.1. Links of Early COVID-19 Cases with Permafrost Distribution

**Figure 1:**
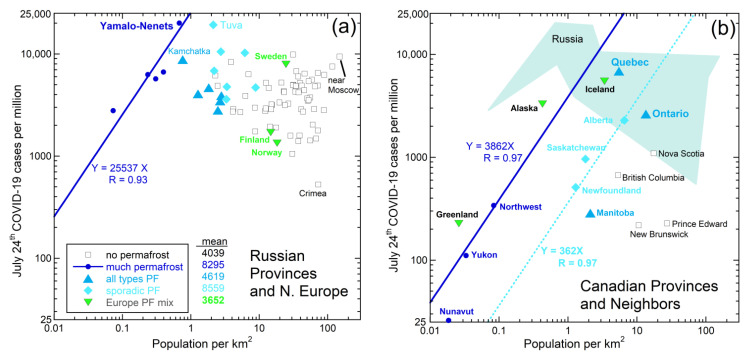
Total cases of COVID-19 in July 2020 for northern regions vs. population density: (**a**) Eurasia, with mean cases per million listed for the various categories; (**b**) North America, with comparison to Russia. Data for both panels from [50,51,52,53,54,55,56]. Regions are grouped by the character and amount of permafrost, based on maps (e.g., [38,52,54]). Because the regions are large and have more than one type, we categorized these as listed in the legend, where all types indicate that the region had continuous, discontinuous, and sporadic PF types. Overall, cases in Eurasia correlate positively with the amount of permafrost and negatively with population density, whereas cases in Canada and nearby correlate positively with the population density and are independent of permafrost coverage. Population is distributed differently with latitude and thus with permafrost in Eurasia and North America. The two most sparsely populated regions in Russia have >150 cases in populations of >41,000, which are statistically significant. The point for Nunavut Province in Canada is a maximum, assuming 1 case in 39,000 people. Currently, 351 cases exist.

Many regions in Russia with permafrost had more COVID-19 cases per million people through July than those without, irrespective of population density (Figure 1a). PF is characterized as continuous (if frozen for >2 years), or by discontinuous, or sporadic as it melts, see, e.g., [47,48,49]. Hence, we categorized regions based on maps of areal coverage of these three types [50,51,52,53,54,55,56]. One region in Russia with much continuous permafrost, Yamalo-Nenets on the Arctic Ocean, was twice as impacted as the US in July, which was then considered high. Yamalo-Nenets is the source of 90% of Russia’s gas and much oil. Industrial activity promotes permafrost melting. Certain regions with sporadic permafrost that is near melting, and some with all of the various PF types, also have a high case incidence (Figure 1a). In particular, the Tuva Republic bordering on Mongolia is encircled by the Sayan and Tannu-Ola ranges. The resulting Tuva depression, which restricts air circulation as demonstrated for the Los Angeles basin (Section 5.4), may locally retain microbes released from its substantial permafrost.

Overall, cases per million in Russia increase with permafrost amount but decrease with population density, which generally is high in regions distant from continuous permafrost. These trends implicate permafrost as the reservoir of the SARS-CoV-2 virus. 

We examined cases as a function of distance from various regions with permafrost, but did not find evidence for a precise geographic center. Nonetheless, much lower case numbers and different trends in Canada (Figure 1b) suggest that Siberian permafrost released the SARS-CoV-2 virus in greater amounts than elsewhere. Roughly, Siberian PF covers 15% of land in the Northern Hemisphere [47]. Canadian permafrost covers much less area (e.g., [38]) and is further from population centers. Very little PF exists in Europe and elsewhere. 

Volume, antiquity, and demonstrated sequestering of viruses (Section 1.1) are evidence for organic-rich permafrost being the source of the three recent coronaviruses. Early COVID-19 case distribution (Figure 1) links to this environment. More recently, two new strains causing the COVID-19 illness were found at high latitudes. One strain, detected in September, is associated with mink farms in Denmark [57]. Another strain motivated closure of London markets in December [58]. 

Although viruses in permafrost have been recently studied [19], these samples were collected in 2015, predating COVID-19, and larger sizes (>220 nm) were examined. Data on small viruses from PF seem currently unavailable. Studying small viruses from regions of PF involved in melting in 2019–2020 might provide valuable information.

### 3.2. Correlations in the Timing of MRVPs, Solar Cycles, and Permafrost Melting

**Figure 2:**
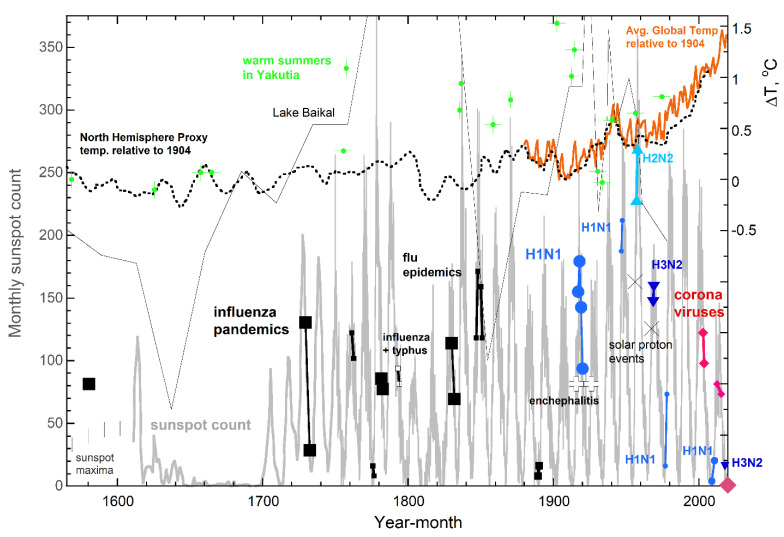
Comparison of pandemic onset and duration with solar phenomena and warming episodes. These correlations that began with the “first” influenza pandemic of 1580. Grey curve (left *y* axis) = sunspot number from [67] and Hoyt and Schatten [60], where vertical bars denote earlier maxima from Wu [63]. X = strong solar cosmic ray events [68,69]. Major pandemics (large symbols) from [1,14] plus substantial or notable epidemics (various small symbols, with labels from [70]) are placed on the sunspot curve to best depict timing. Sizes of the symbols suggest severity, e.g., the 1889 pandemic was mild. The earlier SARS and MERS are actually epidemics, but are included, being related to the ongoing pandemic. Orange solid curve (right axis) = the global temperature difference from NOAA [71]. Dotted curve = decadal averages for the NH [66]. Thin black curve = summer temperatures from insects in lake sediments near Lake Baikal close to Mongolia [64]. All curves assume ΔT is 0 °C at 1904. Green + = hottest summers within 600 years from analysis of tree ring growth near the edge of permafrost [65]; vertical placement was ascertained by subtracting 10.5 °C from values in their tables, which reference value was estimated as the 1904 actual temperature in Yakutia from [65] (Figure 5).

During the Little Ice Age [59], which is associated with the Maunder sunspot minimum of 1645 to 1715 (e.g., [60]), influenza pandemics were absent (Figure 2). Solar maxima should promote permafrost melting, because ice strongly absorbs UV (e.g., [61]) and variations in the ultraviolet (UV) spectral region quantitatively correlate with monthly sunspot count, despite UV constituting only ~8% of solar emissions [62,63]. Frequent correlations of hot summers in Siberia with sunspot maxima (Figure 2) provide support.

Based on the above, onset of all five major influenza pandemics [1,14] near or slightly after a monthly sunspot maximum (Figure 2) implicates that permafrost melting released influenza viruses that were “novel” at that time. The importance of solar UV is underscored by an MRVP not following the temperature peak of 1938–1944 that stems from heavy industrialization preceding and during World War II, whereas the horrendous Spanish flu was preceded by a peak in average global temperature (Figure 2) plus hot summers in Siberia [64,65]. Hot spells from 1912–1914 are consistent with some evidence for Spanish flu beginning before 1918 [1,14]. Regarding MRVPs earlier than 1890, temperature curves are decadal averages (e.g., for the NH [66]). However, because this information does not indicate which Arctic summers were hot, it is not possible to further explore this connection. Similarly, records of zombie fires only go back approximately 20 years.

The climate during the ongoing pandemic is unusual because Arctic temperatures are now setting records: these are climbing ~3 times faster that the average global rate and are inducing copious melting [16]. In addition, record numbers of wildfires with underground smoldering over winter occurred in 2019 and 2020 (Section 1.3). Thus, an increase in solar activity seems currently unnecessary for permafrost to melt substantially and release buried viruses.

**Figure 3:**
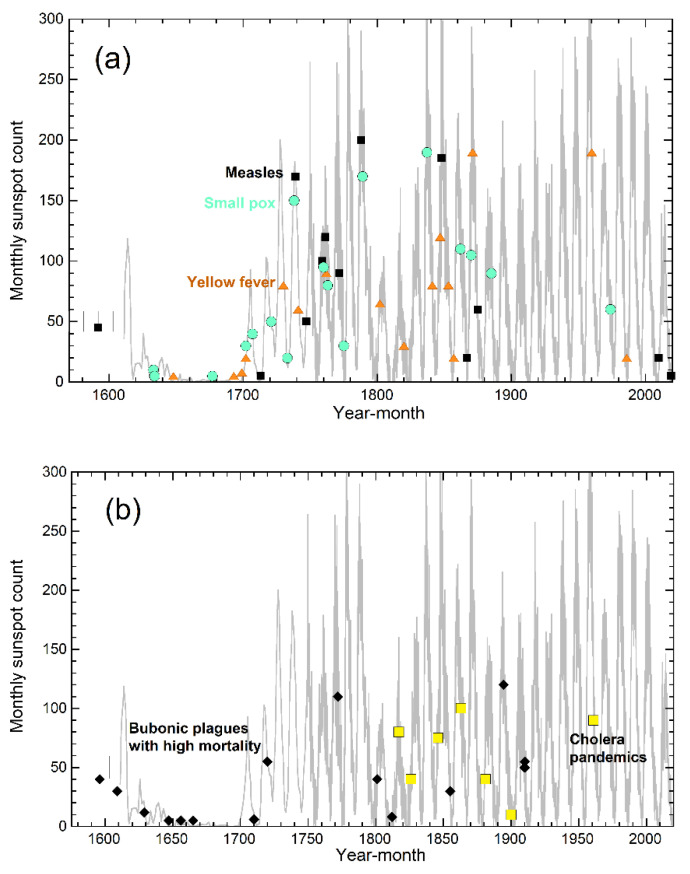
Graphs showing that the onset of various other epidemics and pandemics since the first influenza pandemic of 1580 do not correlate with sunspot number. Data from [67,70]: (**a**) Diseases carried by viruses. Small measles epidemics are included. Historical measles virus epidemics are absent in the Maunder minimum, and somewhat correlate with sunspot maxima, but the scatter is large. Both measles (square symbols) and smallpox (circles) are passed by close contact, whereas yellow fever (triangles) is carried by insects; (**b**) Bacterial diseases. Only major bubonic plagues are shown (diamonds), defined by deaths exceeding 50,000 long ago and 100,000 recently. Cholera (large squares) is water-borne whereas bubonic plague has been connected with fleas.

No such UV correlation exists for the measles virus (Figure 3a), which underlies epidemics only and is inactivated by visible sunlight [72] which is both profuse and apparently constant. Neither do correlations exist with pandemics transmitted by insects or fluids, nor those involving bacteria (Figure 3a,b).

Modern virology studies show that novel influenza pandemics link to high solar activity, whereas rekindled influenzas are associated with minima (Figure 2). The population is more resilient against rekindled viruses, which is consistent with pre-1900 events at minima having lower impact (e.g., in 1889 [2]). Rekindling neither requires novel viruses nor substantial melting of permafrost.

Interestingly, major changes in type A influenza in 1957 and 1968 followed, respectively, the most intense cosmic ray blast from the sun on record [68] and another ground level event of lesser intensity, but longer duration, that was accompanied by a coronal mass ejection [69]. This flux of protons enters at the magnetic poles and so could potentially affect surficial layers of permafrost, either releasing viruses or perhaps inducing mutations via radiation damage.

These findings suggest that typical melting of permafrost each summer replenishes the supply of viruses already in circulation. In contrast, atypically strong melting yields novel varieties. Several hot summers in a row promote upward migration of deep, old organics and microbes along with the sublimated vapor.

### 3.3. Why Pandemics Rarely Onset in Arctic Regions

An interesting question is why emergence is not strongly linked to Arctic regions. An exception is the 1889 influenza linked to St. Petersburg, Russia, which lies 6 degrees south of the Arctic circle. Today the distance to PF is ~1000 km, but western Siberian PF, which is near St Petersburg, is very rapidly melting [48,49]. The first case was traced to October 27th 1889 [2], which is early in the flu season. Tracing of cases via physician’s records over-represents cities, while remote cases would go unnoticed. Based on COVID-19, sudden development of a large number of cases in a populated area precedes spreading of the pandemic. This observation is included in our model (Section 6).

Thus, sparse populations in the Arctic do no provide amplification (see Section 5.3). Possibly, Artic peoples have some immunity from long-term exposure to melting PF.

Regarding COVID-19, it is now recognized that the virus and a few cases existed in December elsewhere than Wuhan (Section 4.2). Whether Arctic regions possess these facets has not been explored to our knowledge.

## 4. Transport of Newly Released Viruses by the Northern Polar Jet Stream

A key issue is the means by which large quantities of virus are transported around the NH in a rather short time frame. Section 1.2 summarizes evidence for long-distance atmospheric transport of viruses. On Earth’s surface, wind participates in not only erosion of rock surfaces, but can transport the scoured particles for long distances before deposition. Volumes are significant: for example, aeolian processes have built the sand dunes across North Africa (e.g., [73]). Winds are both local and global. Regarding the NH, the Jet Stream is arguably the most prominent global wind pattern. The Jet Stream overlies the seasonally variable, vertical intersection of the Polar and mid-latitude atmospheric circulation cells and is at the top of the troposphere, below which the live virus-containing organic aerosols lie (e.g., [4]).

### 4.1. Emergence of COVID-19

Distribution of early cases contain information on how the virus is conveyed after release, proposed to be from PF. Data are contained in case reports, e.g., [74], and have been archived [44,45,46]. For parallelism with historic influenzas, we first consider ~50 cases per day in any given country as indicating emergence.

**Figure 4:**
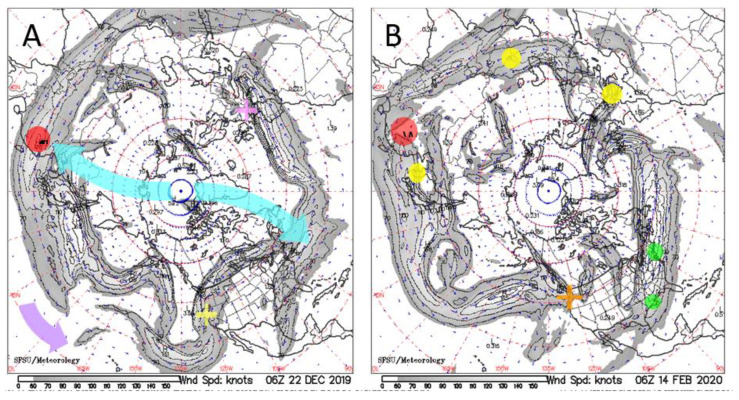
COVID-19 onsets [46,74] compared to NH Jet stream behavior. Maps from California Regional weather server [78]: (**A**) 22 December 2019. Red dot = Wuhan, China. Purple arrow = W to E flow of Jet stream meanders and winds. Blue arrows = sketch of surface winds in the Polar cell, which flow South and slightly West according to the Coriolis effect, rise under the Jet stream, return North near the top of the troposphere, and sink at the Pole. Flow towards France is consistent with discovery of a single case in December (pink cross), as rainout was into the Atlantic and likewise for the Pacific (bottom arrow), so isolated cases appeared in February in the US (yellow cross); (**B**) 14 February 2020. Yellow dots mark Italy, Iran, and Korea, which gained substantial cases in February. Green ovals mark New York City (under strong winds) and New Orleans with climbs in early March. Orange cross marks 37 deaths in February in a Seattle, WA nursing home.

COVID-19 onset during North Hemisphere (NH) flu season at various geographic locations, all of which were frequently beneath the Northern Polar Jet stream from December 2019 through March 2020 (Figure 4). Although air flow in the Jet Stream itself is fast, the meandering pattern of this river of air near the top of the troposphere changes slowly. Near winter solstice, the Northern Polar Jet Stream is strong and reaches low latitudes, including 30° N which crosses Wuhan, China (Figure 4A). After the Jet stream travels east over cold Tibet, it intersects moist warm air from seas south of China, producing rain. For the entirety of December 2019, Wuhan had fog, rain, or clouds every day, as is typical for this winter month [75,76]. For comparison, St. Louis, Missouri in the middle of the USA which had similar temperatures, but no overhanging Jet stream, and was cloudy only ~½ of December with rain once, had negligible cases until spring 2020, and a low incidence compared to neighboring states up to September 2020, despite 3 million people living in the greater St Louis metropolitan area.

By late January, COVID-19 was detected in 25 countries. Cases climbed by February 22 in Korea, Iran, and Italy. This sequence correlates with Jet Stream behavior. From mid-January to early February, the Jet Stream passed over Northern Italy and Iran (Figure 4B). These are Iran’s rainy months. The other countries have large bodies of water to the west or south or both, which bring in moisture. All are North of Wuhan’s latitude. Later emergence further north is consistent with contraction of the average Polar cell, which is accompanied by precipitation progressively changing from snow to rain at any given Northern latitude location, as spring approaches.

During winter 2020 Jet Stream flow towards New York City (NYC) was often from both Canada and the Gulf of Texas (Figure 4A). Cold NYC was greatly impacted by early March, and remained so for most of the spring (Figure 4B). New Orleans was also strongly impacted early on. This notably humid city was also commonly under the Jet stream. Likewise, a high incidence exists in the damp UK. This observation, plus France being impacted much more than its neighbor to the west, Germany, which is further from the Atlantic, point to the important role of rain under the Northern Polar Jet stream as its meanders moved eastward and northward around the globe during development of COVID-19. The role of rain in delivering the virus and widespread initial distribution are suggested by its detection in Northern Italian wastewater samples from December 2019 to February 2020 [77].

Regarding the Arctic, the polar cell is small and the Jet Stream is weak to non-existent when PF is melting in mid-July [78]. Wildfires [35] could carry the virus upwards. As the Jet Stream develops and strengthens over the fall and winter, it moves further south. Deposition via rain could occur in the Arctic summers, but pandemics apparently emerge in large cities. These are distant from permafrost (Section 3.3).

### 4.2. Genomic and Other Evidence for Multiple Points of Origin

Regarding “first” cases, a tightly constrained point of origin does not exist as evidence for the Wuhan wet market and laboratory origins is not convincing [7]. Elsewhere, early cases exist that are unconnected with airplane travel, e.g., one case in France in December [79], and four in rainy areas of the US west coast from January to February [80]. Historic influenza pandemics, despite emergence being evaluated in retrospect and mostly pinned to large numbers of infections, behave similarly. Several countries were infected at different times, plus the same region of the globe was involved in many different MRVPs [1,2,14]. Emergence occurring frequently near China is consistent with weather patterns being repetitious (see below).

Difficulties in deducing a unique point of origin for historic influenzas is consistent with the dataset on the SARS-CoV-2 genome. Initially, Tang et al. [81] showed this coronavirus is distinct, and that two types existed, although both types infected one person from New York City. A highly publicized subsequent finding was that types in Europe and China differed. However, the now vast dataset on genomic variations shows that various types found in 2020 are distributed around the globe [82]. Furthermore, individual patients have four variants on average [83]. Previous interpretation of these genomic variations as mutation with time assumes a “first case” and that the virus changes it transfers among humans. Small changes in the virus are well documented (e.g., [84]), but no intermediate between the inferred precursor virus and SARS-CoV-2 has been found [20]. Alternatively, the virus originated in some area or areas of permafrost, where it may have mutated over millennia since burial, or it may have been initially deposited as many varieties. In either case, whatever viruses were released in the most recent, hot Siberian summers with record numbers of wildfires (e.g., [37]) apparently were then mixed in autumn Northern Polar circulation.

Release from permafrost is supported by recent developments. (1) The September 2020 infections in Denmark associated with mink farms, which followed record Siberian PF melting in July, involved a strain different from those detected the previous winter [57]. By late August, the Polar circulation cell had grown to include Denmark. (2) A new variant further south in London provided a significant number of infections to close holiday markets and restrict travel in December 2020 [58]. This strain appeared earlier in the fall, as did (3) a variant in California, with one case tracing to July [84]. Manifestation of the virus as different varieties whose proportions vary with the timeline of the pandemic is consistent with increased melting of PF with each succeeding summer.

## 5. Environmental and Human Factors Propelling COVID-19 Growth

**Figure 5:**
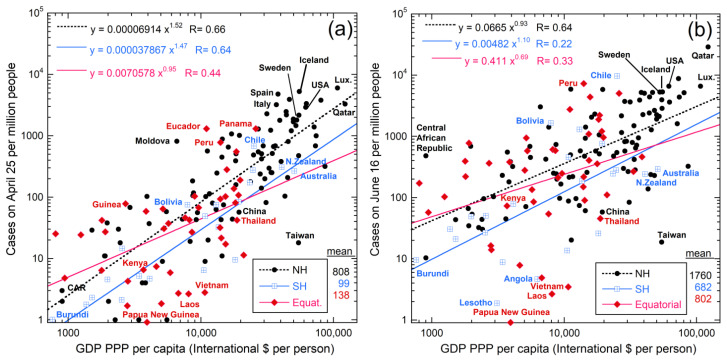
Log–log plot of incidence as a function of wealth of each nation, excluding city-states, small island nations and island dependencies. Data from [53,85,86,87]. To remove possible effects of regions with extremes of wealth or population density, we culled the city-states, countries with areas below ~800 km^2^ based on [87], plus Greenland and the Falkland Islands, which are economic dependencies and are populated only along their coasts. Categories and mean values are shown in the lower right corner. The equatorial region includes countries near the equator that possess substantial (>~40%) areas of hot and wet climate zones (Af or Am classifications; see, e.g., [88]), approximately 10° N to 10° S. Upper left corner shows power-law least-squares fits with linear correlation coefficients (R). Varied growth in regions and certain countries is evident in the labels. The changes in the SH are connected with the Andean nations, despite isolation of mountainous areas: (**a**) case data from 25 April 2020; (**b**) case data from 16 June 2020. Growth in the equatorial regions was strong over the spring.

To help distinguish natural from human factors, we compare COVID-19 cases per million people per country (denoted “incidence”). Comparing 25 April to 16 June data in Figure 5 yields information on the mostly natural spread of the disease during spring 2020, since travel restrictions were in place at that time.

### 5.1. Links with Latitude and Climate

On 25 April, the NH had a 7-fold higher incidence than countries in the equatorial belt, and an 8-fold greater incidence than in the Southern Hemisphere (SH). Three categories were considered (Figure 5), since NH equatorial regions have much warmer climates than Europe. Recasting this climatological division to a purely latitudinal division (e.g., ±10°) changes the categories of a few countries but does not substantially alter these geographic differences. Additionally, a NH excess remains if only the two hemispheres are considered.

From 25 April to 16 June, incidences grew, but SH growth rate outpaced the NH, so the NH/SH ratio was reduced from 8-fold to 2-fold. If human factors are the same in the three regions, then SH growth in spring reflects natural causes, since international travel had been severely restricted at that time.

Limited communication of atmospheric circulation between the NH and SH was demonstrated long ago by tritium distributions after above ground nuclear detonations. However, detonations are instantaneous, whereas infections develop over weeks while weather patterns change. Thus, NH-SH communication of air during pandemic development is expected. Hence, to ascertain what environmental factors are involved in COVID-19 spread from April to June, we plotted the data in various ways. We found that the incidence within each of the three latitudinal divisions depends on wealth, as quantified by the gross domestic product based on purchasing power parity (GDP-PPP) per capita (Figure 5). These nearly parallel hemispheric trends support that human factors are similar overall. A slightly weaker correlation of incidence with median population age exists (not shown), but median age is linked to wealth. Incidence correlating with wealth is attributed to the number of confirmed cases depending to the number of tests conducted, which in turn depends on economics of the country. The data are more reliable for wealthy countries, where tests conducted early on greatly exceeded infections. We did not find a correlation with population density.

**Figure 6:**
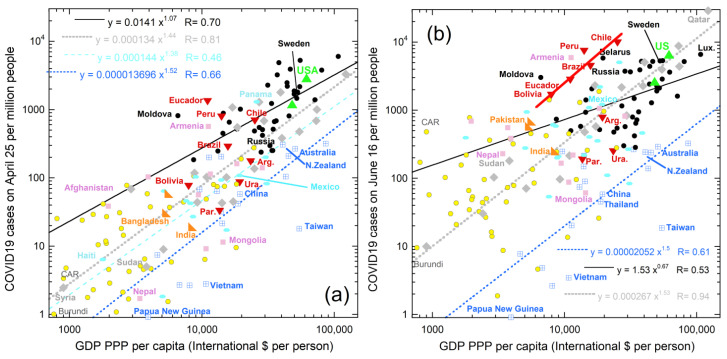
Log–log plot of incidence for proximal countries vs. wealth. See Figure 5 for data sources and text for groupings of countries: (**a**) COVID-19 on 25 April 2020; (**b**) COVID-19 from 16th June. Data from [46,53]. Changing categories of a few countries little affects the fits: examples are transferring Russia from the Europe grouping (black dots) to Central Asia (pink squares); or Mongolia to Southeast Asia/Oceania group (blue crossed squares); or Sudan from the Middle East (grey diamonds) to Africa (yellow dots with rims). Other symbols are: Red down-pointing triangles = South America countries which lie mostly in the SH. Turquoise oval = Mexico, Central American, and the Caribbean. Green up-pointing triangles = USA and Canada. Orange right triangles = populous India, Bangladesh, and Pakistan. The best constrained fits are shown.

Incidence also depends on wealth for geographic groupings (Figure 6), which divisions are based on continents and climate. Mexico is much warmer that the US and Canada and many of Mexico’s population centers are in its southern regions. Hence, Mexico was included with the Caribbean and Central Americas, which are close to the equator, but north of it. For consistency in grouping, the South America category includes only countries with large tracts in the Southern Hemisphere. Russia’s population is mostly near Europe and is grouped with these countries due to similarly high latitude. India, Pakistan, and Bangladesh were grouped together due to proximity, having ocean to the south, and very dense populations. The Central Asia group is limited to land-locked countries (neglecting existence of the Baltic and Caspian Seas). Southeast Asian countries are grouped with Oceana due to all have substantial coastlines and fairly high populations. The Middle East is singled out due to this being a northern region that is hot and dry, which differs from characteristics of the other groupings. Africa was not further subdivided due to poor statistics of early COVID-19 cases.

Trends being parallel in April (Figure 6a) results from onset depending on latitude and its initiation in the NH. Data from June (Figure 6b) shows that the incidence of COVID-19 increased everywhere except in the S.E. Asia and Oceania countries. Strong increases occurred on the west coast of South America, and somewhat in South Africa. The winter flu season is not the sole cause of these SH increases, since growth in Australia and New Zealand was small. Global weather patterns and climate, which are largely but not entirely latitudinal, are implicated; see the Koppen-Geiger maps [88]. Countries with oceans to the west differ from those with oceans to the east, reiterating the importance of rain and prevailing winds that were indicated in Figure 2. Because a key driver of weather and climate is sunshine, solar variations are further explored below.

### 5.2. Links with Solar Irradiance

Sunlight received depends mostly on latitude, but not entirely. Global Horizontal Irradiance (GHI) from the Global Solar Atlas [89] was used to quantify this factor because this sums direct irradiance (after accounting for the solar zenith angle) with diffuse horizontal irradiance.

**Figure 7:**
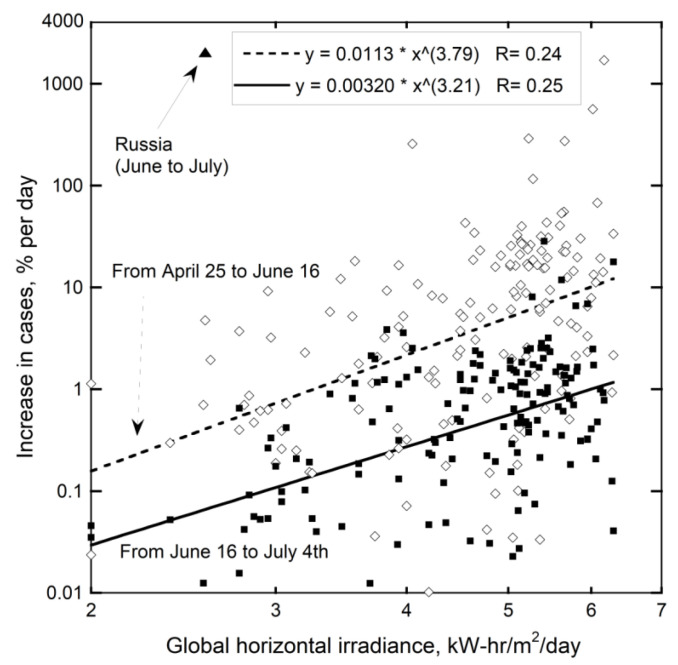
Logarithmic plot demonstrating that case increases per day, for each the spring and summer correlate with median total solar irradiance. COVID-19 data from [46,53]. The maximum and minimum GHI reported for each country on the Global Solar Atlas website [89] were averaged. Diamonds = all countries over late spring. Filled squares = increase over the summer solstice. The extreme increase in Russia occurred just after the record-breaking temperature highs in Siberia in June.

**Figure 8:**
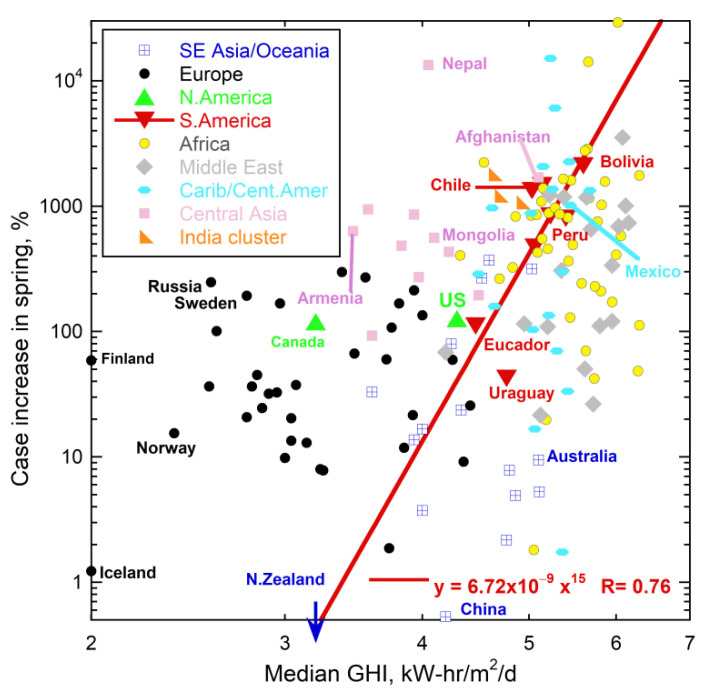
Increase in COVID-19 cases listed in [46,53] in the spring as a function of solar irradiance from the Global Solar Atlas website [89], averaged over the year. Countries grouped as in Figure 6. New Zealand cases decreased, as also occurred in France (not shown). A few counties with 1–3 cases were excluded to reduce noise. GHI was estimated for the northernmost countries, which include Canada and those near the left axis, with labels, by assuming their lowest measured GHI value was 2. Russia, Sweden, and Finland, which are the three countries nearest to Siberian permafrost, all have high case increases for their GHI values. Similarly, Canada which has substantial permafrost has a large number of cases given its irradiance. Unlike Figure 7, a per day basis was not used, and only the April-to-June increase is shown.

Over the spring and near the summer solstice, the increase in cases in the various countries roughly depends on their median solar input (Figure 7). Nations with high GHI are in Africa, the Middle East, or contain the Andes, where GHI of the latter is augmented by high altitude. High GHI exists in Mexico, Afghanistan, parts of Brazil, and the southernmost US (the Sunbelt). These regions show strong increases in spring and early summer (Figure 5, Figure 6, Figure 7 and Figure 8). Nepal likewise has a strong increase, due to UV radiation increasing with elevation as the atmosphere thins. Spring increases are generally higher in the NH than in the SH for countries with similar GHI, which is consistent with GHI increasing in the NH spring. Early summer growth in Europe occurred in its sunniest nation, Spain. In the SH, COVID-19 cases increased in Australia in August, as the SH spring begins, but not in New Zealand, which is far less sunny.

Cases increased substantially in Russia near summer solstice (Figure 7). Changes in the solar zenith angle are not the reason, because nearby nations show no such a strong increase. Importantly, on 20 June 2020 record temperatures were set in Siberia, which broke the sequential records of 2019 and 2018. From this data and trends in Figure 1, more coronaviruses were released from the permafrost this summer, which release affected humans near the source. The 2020 release explains the appearance of new varieties in Denmark and the UK, discussed above, as well as rapid spreading of the UK variant, despite reduced travel in and between countries during 2020.

### 5.3. Human Immune Response to UV

Historic MRVPs occurred during sunspot maxima, when solar UV radiation is at its peak (Figure 2). COVID-19 cases grew in regions as their GHI increased substantially (Figure 5, Figure 6, Figure 7 and Figure 8). Both correlations are attributable to an increase in UV inducing immunosuppression [39,40,41,42,43]. In detail, solar UV dampens the production and development of T-cells (CD4+ CD25+ foxp3+ cells) as well as activating regulatory B-cells which further suppress activation of T-cells [90,91,92]. High human sensitivity to UV is evidenced by deaths from pneumonia fluctuating with sunspots [93]. Our findings are supported by the fact that influenza pandemics caused by novel viruses occurred during the summer when the NH would be receiving above average solar radiation. Continuation into autumn reflects that incubation and spread require finite time.

Influenzas which arise during local minima of sunspot activity tend to involve viruses that resurfaced from previous pandemics (Figure 2) and in all cases emerge during the autumn and winter months. This can be explained by a lack of vitamin D hindering activation of the immune system. Indeed, there is a growing body of evidence that vitamin D can be immunosuppressive if its quantity is either too low or too high [93,94]. The traditional Chinese diet lacking dairy is likely low in vitamin D and may have promoted winter emergence in Wuhan.

Interestingly, studies evaluating the effect of UV exposure on COVID-19 cases up to 10 April [95] and up to 8 May [96] show that in the spring UV is beneficial. These findings are consistent with low to moderate exposure having a beneficial effect on immunity. Similarly, in the spring supplementation of vitamin D, which is produced during UV exposure, was argued as beneficial, with dose being a consideration [97]. However, vitamin D was no longer recommended by summertime [98]. These results are consistent with certain ranges of UV and vitamin D each being beneficial to immune response, whereas shortages and excesses have converse effects [93,94].

These observations strongly suggest that pandemics spread most easily when the general populace is experiencing suppressed immune systems. This does not require all people to have suppressed immune systems, rather only a subset of the population with weak immune systems allows infections to spread faster and farther than these otherwise would. Thus, human UV response provides a means for runaway growth when UV is either very low or very high.

### 5.4. Links with Poor 2020 Air Quality in Los Angeles

Air quality in the Los Angeles basin is naturally poor, due to wind patterns and being encircled by mountains, which problems are exacerbated by its large human population [99]. The notable surge of COVID-19 cases late in 2020 [100] follows the worst smog event in the greater Los Angeles area since the 1990s. From the summary of Barbosa [101], ozone levels exceeded federal health standards for 157 days and levels of particulates were in the bad range for 30 days. Contributing factors are the extreme heat waves in the summer of 2020 elevating ozone levels whereas significant wildfires in the fall produced record-setting levels of soot.

From Section 1.4, COVID-19 cases elsewhere correlate with particulates in the air. Concentration of smog near the ground in the cooler weather explains the recent winter surge in Los Angeles area.

Ozone levels are independent of particulate load. Although particulate matter apparently poses the greater danger to human health [31], which is attributable to their variety of sizes and chemical compositions, formation of free radicals by ozone is considered to cause inflammatory injury to lungs [102]. Uptake of ozone gas is not affected by masks. People with pre-existing respiratory disease are more vulnerable, as evidenced by elevated ozone levels causing increases in hospitalizations for respiratory illnesses, such as asthma [103]. As noted in Section 5.3, part of the population being affected can result in a runaway response.

## 6. A Model for Natural Causes of Major Respiratory Virus Pandemics

Table 1 summarizes our model. We propose that novel viruses released during summer permafrost melting, being tiny, are picked up by low speed winds as aerosols [4], to then circulate in the contracted North Polar atmospheric cell. Wildfires associated with these hot summers provide updrafts, thereby aiding ascent of the microbes. Moreover, the consequent subsurface fires smoldering over winter should promote subsurface PF melting. Cold storage preserves viruses both in PF and airborne while high latitudes and the organic aerosols protect the viruses against inactivation via solar UV.

In the fall, the North Polar cell expands, reaching its lowest latitudes mid-winter. Where warm moist air at its southern boundary is intersected, rain deposits suspended viruses on Earth’s surface, analogous to previous observations of viruses in dust storms [104]. Particulate content of the air correlating with case load (Section 1.3) supports this contention.

When virus quantities are large, and immunities are low to the particular viral crop, a major pandemic can start. COVID-19 began at the beginning of flu season, when people were vulnerable, and in densely populated regions of China, promoting multiplication via human interaction. When it is not raining in the initial winter stages, atmospheric circulation likely pulls aerosolized virus up from the infected area, back towards the North pole and down again, adding to atmospheric concentrations in the southbound surface flow. Rainouts later (e.g., February 2020) occur globally, progressing increasingly northwards, while depending on specific Jet stream configurations.

In late spring to summer, we propose that UV suppression amplifies the cases in sunny regions. Due to this amplification, historic pandemics of novel viruses are traced to summer and autumn. This deduction is consistent with summer amplification of COVID-19 in the southern part of the NH. Reoccurring viruses require longer for the UV human response to produce a large number cases. Hence, historic rekindled MRVPs appear in fall to winter, when humans are most vulnerable. The solar cycle contributes, as described above, but the launch of viruses requires a “hot” Artic summer. Yamalo-Nenets permafrost (PF) could be a reservoir of SARS-CoV-2 virus, but, more likely, many tracts of PF which were deposited at some certain point in time, and are now melting, are the source. The facts that Siberia has the largest area of PF, and that Russia experienced a jump in cases just two weeks after record (100 °F) temperatures, support this deduction.

**Figure 9:**
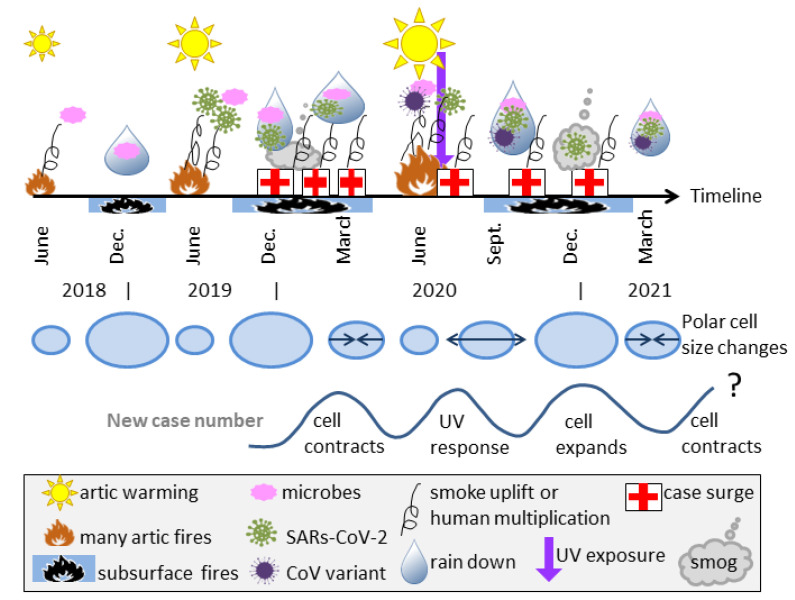
Schematic of the proposed time evolution for COVID-19 in the Northern Hemisphere. The date line is non-linear and approximate. Top row of symbols shows the inferred timing of various natural processes, where the symbols are described in the legend (grey box at bottom). Middle row of blue ovals shows the sizes of the North Polar cell connected with seasons, and whether it is expanding or contracting (arrows). Grey curve shows the wavelike number of case increases and how these are connected with the Polar cell cycles and human UV response. However, the UV peak is only expected for areas with seasonal UV changes. In our model, cyclically occurring natural processes both lead up to the pandemic and promote its longevity. Major pandemics are rare because once any given region of permafrost is melted and releases its particular microbes, this locality is depleted.

Historically, major pandemics persisted several years in a row. Possible causes are recirculation of incubated viruses as the Polar cell cyclically contracts and expands, or additional release of similar viruses during several “hot” Arctic summers in a row. Both possibly describe waves in the Spanish Flu: however, genetic tests postdate the infections, so quantification is unclear. Figure 9 provides a possible explanation for the wavelike nature of cases in MRVPs from data on COVID-19. For example, the summer jump of COVID-19 cases in Russia and the September event near Denmark mink farms, which was followed by another strain variant in London suggests 2020 release of additional virus types, which implies continuation of the pandemic beyond the present, just as the Spanish Flu strain is related to all subsequent influenzas. Impact in the future depends on many factors (Section 7).

## 7. Model Predictions and Tests

In the past, MRVPs were fortunately infrequent because many factors were required (Table 1). Today’s immense human population alone provides unprecedented amplification. Current preventative measures therefore focus on human-to-human transmission. A factor worth considering is seasonality, which is out of phase in the Northern and Southern Hemispheres. Combatting UV immunosuppression during high influx can be achieved through use of sunscreen or protective gear. Information on sunscreen use of February 2020 vacationers in Italy and Florida may be useful in ascertaining the importance of UV suppression. Notably, in the spring, when exposure to sunlight was low, use of vitamin D appeared to be helpful [97], but studies later in the year provided equivocal evidence (e.g., [98]). Levels of UV exposure, dosage of vitamin D, and their interrelationship may need to be considered in evaluating the role of immunosuppression in pandemic development and possible means of mitigation. Another possible factor is use of air conditioners, given the findings of Mathai et al. [12], since the moist environment in these devices may permit virus survival. Use of air conditioning is fairly recent and prevalent in wealthy localities with hot summers, such as Phoenix, New Orleans, and Los Angeles, all of which were impacted significantly.

Humans move indoors during winter, along with the virus. Airborne transmission in closed places can be combatted using air filtration, which is also testable. Sufficiently impeding circulation of small virus particles may require improved technology, as filter tests are geared for larger sizes, due to difficulties in measuring particles with sizes similar to wavelengths of UV light. Circulation between apartments in large housing facilities may promote spreading over modest distances, which can be evaluated.

Record-setting summer maximum temperatures for three consecutive years in Siberia, accompanied by surface and zombie fires, suggest that more viruses were released, and have been stored in the North polar cell. This is testable. Permafrost samples are cold-stored in various laboratories. Documentation of collection dates and localities should exist. The presence of coronaviruses in 2019 samples, but low counts or absence in earlier years, would confirm a key component of our hypothesis. However, due to the vast tracks of PF in Siberia, it is possible that the miniscule laboratory samples do not include source material.

Although areas of permafrost melting could be tested for virus content, this may be haphazard. Other possibilities exist. One is to collect and test atmospheric samples as a function of latitude and geography, after Setti et al. [25], who detected RNA from the coronavirus in the atmosphere of Bergamo, Italy. Population concentrations are likely important. The Los Angeles basin, and similar geographical areas, may trap the viruses, including those muliplied by humans, as discussed in Section 5.4. High case numbers from the Tuva depression of Russia support this contention (Figure 1a) as do the many studies discussed in Section 1.2 and Section 1.3, which show that wind geography and pollution are important factors in the airborne spreading of small viruses.

Another possible test would be to compare the Jet stream pattern in the NH as fall and winter progress against development of COVID-19 cases. Data on the US qualitatively indicate a southward projection, as elevation of case numbers in St. Louis this November were preceded by surges to the north, and then was followed by surges in Los Angeles further south. A thorough study may provide a means for short-term prediction.

The unique aspect of the current pandemic is its strong impact on South America. Of great relevance to understanding MRVP development is amplification of airborne virus by infections in countries south of the equator. New Zealand has mostly eliminated the virus, and has low GHI. Resurgence there would demonstrate global transport by the Southern Polar atmospheric cell, although we think this is unlikely.

However, our main concern is that escalating melting of Artic permafrost, if left unchecked, may resurrect another, more virulent agent in the summers to come. A reduction in human contributions to Arctic warming is essential, as has been argued by many for other reasons.

Fall 2020 upswings of cases [46,53] occur in high northern latitude countries (e.g., Russia, Denmark), whereas nations near the equator now show flat case numbers (e.g., Brazil). These trends are consistent with fall growth of the Polar atmospheric cell increasing airborne virus concentrations in the NH at high latitudes. Measuring the latitudinal dependence of virus concentrations in the air could quantify the role of permafrost in replenishment during prolonging pandemics, as well as the role of atmospheric transport. However, human factors are becoming increasingly important.

Snow’s circa 1850 recognition that cholera was water-borne preceded eliminating these pandemics. The source of this bacterium predates his London investigation, and its origin is not known. Perhaps the COVID-19 pandemic did begin with a few rare bats from China, although no route of transmission is evident including via the researchers and laboratory involved [8,9,10], the quantity of virus seems grossly inadequate to impact the large city of Wuhan with 10 million people, and the multiple bat virus locations [20,21] are not consistent with precipitous, geographically restricted emergence. Notably, bats fly in air and roost together in damp, closed caves, and so could be infected by an airborne virus similar to SARS-CoV-2. Likewise, the ~million minks on the Denmark farms are exposed to the air and are clustered together, although this is not a natural setting for these mammals. Known, contemporaneous animal infections involve the SARS-CoV-2 virus, not a precursor (e.g., [105]). Transmissions occur where humans exist. Whether the route requires direct contact with an infected human or is indirect, via a virus-laden atmosphere, is testable. The San Diego gorillas have outside access whereas the zookeepers were described as asymptomatic, and to our knowledge primates indoors, as in St. Louis, have not been infected, but would similarly have some contact with caregivers.

Contemporaneous infection and spotty deposition by air currents appear to describe continued developments of COVID-19. Knowing where the virus originated would be useful for mitigation, but permafrost, proposed as the physical origin here, is huge and so pinpointing is questionable. Atmospheric transport playing the key role over moderate to large length scales warrants further investigation, and such will hopefully lead to mitigating actions. Last, but not least, the role of ozone needs further investigation, given developments in Los Angeles and previously established exacerbation of respiratory illnesses with high ozone levels.

## 8. Conclusions

Release of stored viruses from melting permafrost has great potential to contribute a large variety of related and unrelated infectious agents [15]. The present paper constructs a model of how environmental processes could assist release from PF and promote global spreading via atmospheric transport in the earliest stages of a MRVP, focusing on the plethora of data on COVID-19 during its developmental stages and its shared features with major influenza pandemics. We explain the hallmark characteristics of semi-regular, but precipitous, appearance approximately every 30 to 50 years in approximately the same region of the globe. These key features are not explained by existing hypotheses of mutated viruses from rare bats or laboratory accidents, both of which have been contested [8,9,10]. In constructing our model, we consider modern studies of genomes and human immune response as well as information on atmospheric transport of viruses and climate change. Table 1 and Figure 9 summarize the proposed steps and time evolution, while Section 7 covers predictions and tests. The pandemic is not over, and human behavior competing with natural causes could alter its future course. We hope that our assessment and findings will improve understanding while providing practical benefits.

## Figures and Tables

**Table 1:** Synopsis of our model linking stages in the development of major respiratory virus pandemics to natural phenomena and environmental processes.

Stage	Natural Process	Key Evidence or Reason
Novel virus source	Permafrost melting	Massive reservoir of ancient infectious agents
Sporadic timing	Climate and solar cycles	Semi-regular; seasonality of flu; rekindling
Virus transport	North Polar Jet stream	Rapid onset at similar locations historically
Virus deposition	Rainout; pollution	Viruses and disease linked to particulates
Case growth	UV immunosuppression	Amplification in sunny regions after flu season
Multi-year duration	Atmospheric circulation	Infected hosts increase virus content in air; more permafrost melting augments supply
Historic cessation	Arctic freeze; immunity	Minimal supply with reduced amplification

## Data Availability

All data were obtained from publicly available sources.
